# Surgical Mouse Model of Large Arterial Stiffening Differentially Impairs Long‐Term and Short‐Term Memory in Male and Female P301S Mice

**DOI:** 10.1002/alz70855_106829

**Published:** 2025-12-24

**Authors:** Caroline E Baggeroer, Peter A Pietri, Fiona E. Harrison, Angela L. Jefferson

**Affiliations:** ^1^ Vanderbilt Brain Institute, Vanderbilt University, Nashville, TN, USA; ^2^ Vanderbilt Memory & Alzheimer's Center, Vanderbilt University Medical Center, Nashville, TN, USA; ^3^ Vanderbilt University, Nashville, TN, USA; ^4^ Department of Medicine, Vanderbilt University Medical Center, Nashville, TN, USA; ^5^ Department of Neurology, Vanderbilt University Medical Center, Nashville, TN, USA; ^6^ Vanderbilt Memory and Alzheimer's Center, Vanderbilt University School of Medicine, Nashville, TN, USA; ^7^ Vanderbilt Brain Institute, Vanderbilt University Medical Center, Nashville, TN, USA

## Abstract

**Background:**

Large arterial stiffness is associated with increased tau phosphorylation and neurodegeneration in humans. However, arterial stiffness often co‐occurs with other age‐related conditions like hypertension, atherosclerosis, and diabetes, making it difficult to distill the underlying mechanisms specifically linking large arterial stiffness to abnormal brain function and Alzheimer's disease (AD) pathology. We leveraged a surgical mouse model of large artery stiffness and used it concurrently with a transgenic AD mouse model of tau pathology to investigate the impact of large artery stiffness on cognition.

**Method:**

5‐month‐old male (*n* = 7) and female (*n* = 8) P301S mice, and male (*n* = 11) and female (*n* = 7) wild‐type littermate controls underwent bilateral carotid calcification surgery, where both carotid arteries were exposed to 0.3 mol/L CaCl_2_ or saline as a control for 20 minutes. Carotid compliance, demonstrated as a change in carotid diameter within a cardiac cycle, was measured *in vivo* using M‐mode Doppler ultrasound imaging before and after surgery. Three weeks post‐surgery, mice underwent behavioral testing, including locomotor activity, elevated zero maze, Morris Water maze, Y‐maze, novel object recognition, and nest building.

**Result:**

CaCl_2_ exposure led to decreased post‐operative compliance of the carotid artery as measured by a significantly decreased change in carotid diameter in both the left and right arteries compared to saline‐treated mice. Wildtype male CaCl_2_‐treated mice displayed an impaired ability to learn the position of the escape platform in the Morris water maze. Female P301S CaCl_2_‐treated mice showed reduced time spent in the target quadrant during the probe trial compared to the saline‐treated P301S and wildtype controls. Female P301S CaCl_2_‐treated mice also spent a reduced amount of time exploring the novel object during a Novel Object Recognition task.

**Conclusion:**

Behavioral data shown here suggest that bilateral carotid calcification impairs spatial learning abilities in wildtype male mice and long‐term memory retention in P301S female mice and may also impair working memory in P301S female mice. The carotid calcification model will further be used to investigate whether the carotid calcification surgery accelerates the development of tau pathology seen in the P301S mice.

